# Sodium channel Na_v_1.8 immunoreactivity in painful human dental pulp

**DOI:** 10.1186/1472-6831-5-5

**Published:** 2005-07-07

**Authors:** T Renton, Y Yiangou, C Plumpton, S Tate, C Bountra, P Anand

**Affiliations:** 1 Department of Oral & Maxillofacial Surgery, Dental Institute, Queen Mary's College, London University, Whitechapel, London UK; 2 Peripheral Neuropathy Unit, Imperial College, Hammersmith Hospital Campus, Du Cane Road, London W12 ONN, UK; 3 GlaxoSmithKline, Medicines Research Centre, Gunnels Wood Road, Stevenage, Hertfordshire SG1 2NY, UK; 4 Neurology-CEDD, GlaxoSmithKline, Third Avenue, Harlow CM19 5AW, UK

## Abstract

**Background:**

The tetrodotoxin-resistant voltage-gated sodium channel Na_v_1.8 (SNS1/PN3) is expressed by nociceptors and may play a role in pain states.

**Methods:**

Using specific antibodies for immunohistochemistry, we studied Na_v_1.8 – immunoreactivity in human dental pulp in relation to the neuronal marker neurofilament. Human tooth pulp was extracted from teeth harvested from a total of twenty-two patients (fourteen without dental pain, eight patients with dental pain).

**Results:**

Fibres immunoreactive for Na_v_1.8, were significantly increased on image analysis in the painful group: median (range) Na_v_1.8 to Neurofilament % area ratio, non-painful 0.059 (0.006–0.24), painful 0.265 (0.13–0.5), *P *= 0.0019.

**Conclusion:**

Na_v_1.8 sodium channels may thus represent a therapeutic target in trigeminal nerve pain states.

## Background

Pain is the most common symptom of diseased tooth pulp, often a result of coronal caries of the tooth, affecting up to 80% of the western population during their lives. The mature human dental pulp is densely innervated with over 900 axons entering the average human premolar tooth [1] that originate from the trigeminal ganglion. The normal pulp seems insensitive to exteroceptive stimuli; however, in pathological states, electrical, thermal, mechanical and chemical stimuli all produce a nociceptive response [2]. Primary and permanent tooth pulps contain 70–90% C-fibres [3], myelinated fibres mostly of the A delta category [3], with few myelinated fibres of the A beta group. The majority of nerve fibres terminate in the coronal region of the pulp, forming a subodontoblast plexus, with 40% terminating in the dentinal tubules close to the odontoblast processes [3]. Strong correlations have been reported between the afferent discharge frequency of human pulp nociceptors and pain levels [4]. Many suggestions have been made for the origin of pulpal pain e.g. pulp inflammation involving several mediators located within the pulp (cholinergic and adrenergic neurotransmitters, prostaglandins and cyclic AMP). However, thus far, no correlation has been established between pain characteristics and histology of the pulp [5,6].

Voltage-gated sodium channels play key roles in the pathophysiology of pain and are distinguished according to their sensitivity to the neurotoxin tetrodotoxin (TTX) as fast-activating TTX-sensitive (TTX-S) channels, or slow-inactivating TTX-resistant channels (TTX-R). The distribution and pathophysiology of these channels, particularly Na_v_1.8, (SNS/PN3) have been the focus of research in pain mechanisms [7]. Recently, antisense treatment blocking this channel reduced neuropathic pain [8]. We have previously described the temporal and spatial distribution of Na_v_1.8 in human sensory neurones [9]; the channels were decreased acutely in sensory cell bodies after spinal cord root avulsion but accumulated in fibres proximal to the site of injury in brachial plexus trunks, and in neuromas. Based on the presence of Na_v_1.8 in predominantly small medium sized neurons in human DRG, it is likely that Na_v_1.8 is present in both A delta and C fibres [9,10].

This study aimed to assess if pulpal pain associated with caries was associated with any change of Na_v_1.8-immunoreactivity within tooth pulp nerve fibres.

## Methods

Patients scheduled for dental extraction at Guy's Dental Institute, London, were included in the study, subsequent to providing consent in accordance with the local research ethics committee.

22 permanent molar teeth about to be extracted were tested, 1 hour prior to extraction, for vitality using an electric pulp tester (analytic technology constant current at the mid-buccal surface of the tooth) and with ethyl chloride to confirm the neural vitality of the dental pulp. A pain history was also collected (existing pain and duration). The patients were divided into two groups, those with existing pain from the tooth (n = 8 patients age range: 40.3 ± 4.0 years) and those with no history of or existing pain (n = 14 patient age range: 37.3 ± 14.6 years). The gender distribution of the groups was M:F 1:1. All the dental pain in this study was attributable to pulpitis due to extensive dental caries of the molar tooth, the duration of pain was 2.9 weeks (range 0.5–8), and the indication for extraction of the non-painful teeth was pericoronitis. All the teeth were removed by standard buccal approach under local or general anaesthesia. Subsequent to the extraction process (lasting less than 5 min), the teeth were sectioned vertically with a water-cooled drill and the pulp lifted out, and specimens immediately snap-frozen at -70°C. Intentional examination of the coronal pulp, including the densely innervated subontoblastic layer, was assisted by careful orientation of the pulp on a marked sterile card. Based on the number of inflammatory cells present, the inflammation was graded in accordance with local histological standard scales, which were mild, moderate or severe.

### Immunohistochemistry

Frozen pulp or nerve were embedded in OCT medium (RA Lamb, London, UK) and sections of 12 μm thaw-mounted onto glass slides pre-coated with poly-L-lysine. Sections were immersion-fixed in fresh 4% paraformaldehyde in phosphate buffered saline (PBS) for 30 min, then endogenous peroxidases blocked by incubation with alcoholic 0.3% hydrogen peroxide for a further 30 min. Sections were incubated overnight with a monoclonal antibody to the neuronal marker neurofilament (Clone 2F11, Dako, Cambridge, U.K., used at a final titre of 1:10, 000) and a polyclonal antibody against the Na_v_1.8 (K107), whose specificity has been described by us previously [9]. Sites of primary antibody attachment were revealed using avidin-biotin peroxidase method (Vector Elite ABC method, Vectastain, Novacastra, Newcastle, UK). Preparations were counterstained in 1% w/v aqueous neutral red to visualise nuclei and photographed with an Olympus photomicroscope. Specificity studies of the Na_v_1.8 antibody (K107), showing positive staining in human DRG neurons, and no staining in pre-absorption experiments using Na_v_1.8 peptide in tooth pulp sections, were performed as previously described [9].

### Image analysis

Na_v_1.8 and neurofilament immunoreactivity in fibres were quantified using computerized image analysis (Seescan Cambridge, UK). Quantification of the data was performed using 12-micron thick serial sections. Images were captured via video link to an Olympus BX50 microscope (×40, objective) and scanned by computer. Setting grey-level detection limits at threshold, highlighted positive immunostaining, and the area of highlighted fibres was obtained as % area of the field scanned. Scanning was performed for a minimum of 5 fields at random per tissue section orientated longitudinally, assessed in a blind fashion; 3 tissue sections from each pulp were analysed, and the mean value from each patient obtained. Results are expressed as the average percentage ratio of the mean Na_v_1.8 to neurofilament reactive fibres in 5 fields.

### Analysis

The Mann Whitney test was used to compare ratios between groups; *P *values less than 0.05 were considered significant.

## Results

**Figure 1:**
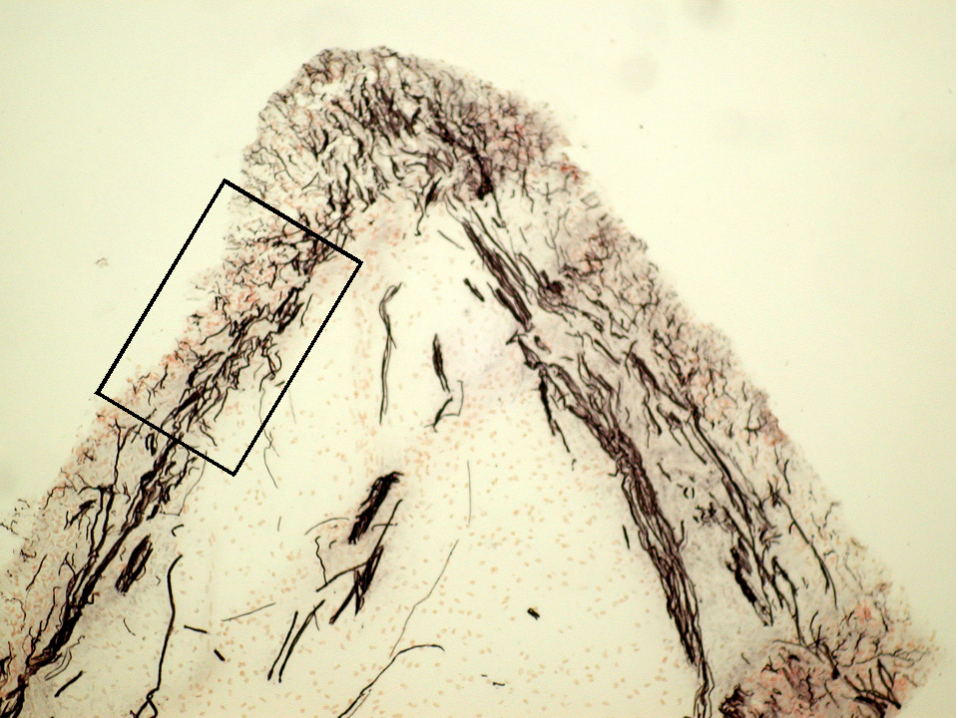
Photomicrograph illustrates the pulpal horn of a molar tooth, highlighting the relationship of the subodontoblastic plexus (boxed area) to the deeper coronal pulp. Magnification × 10

**Figure 2:**
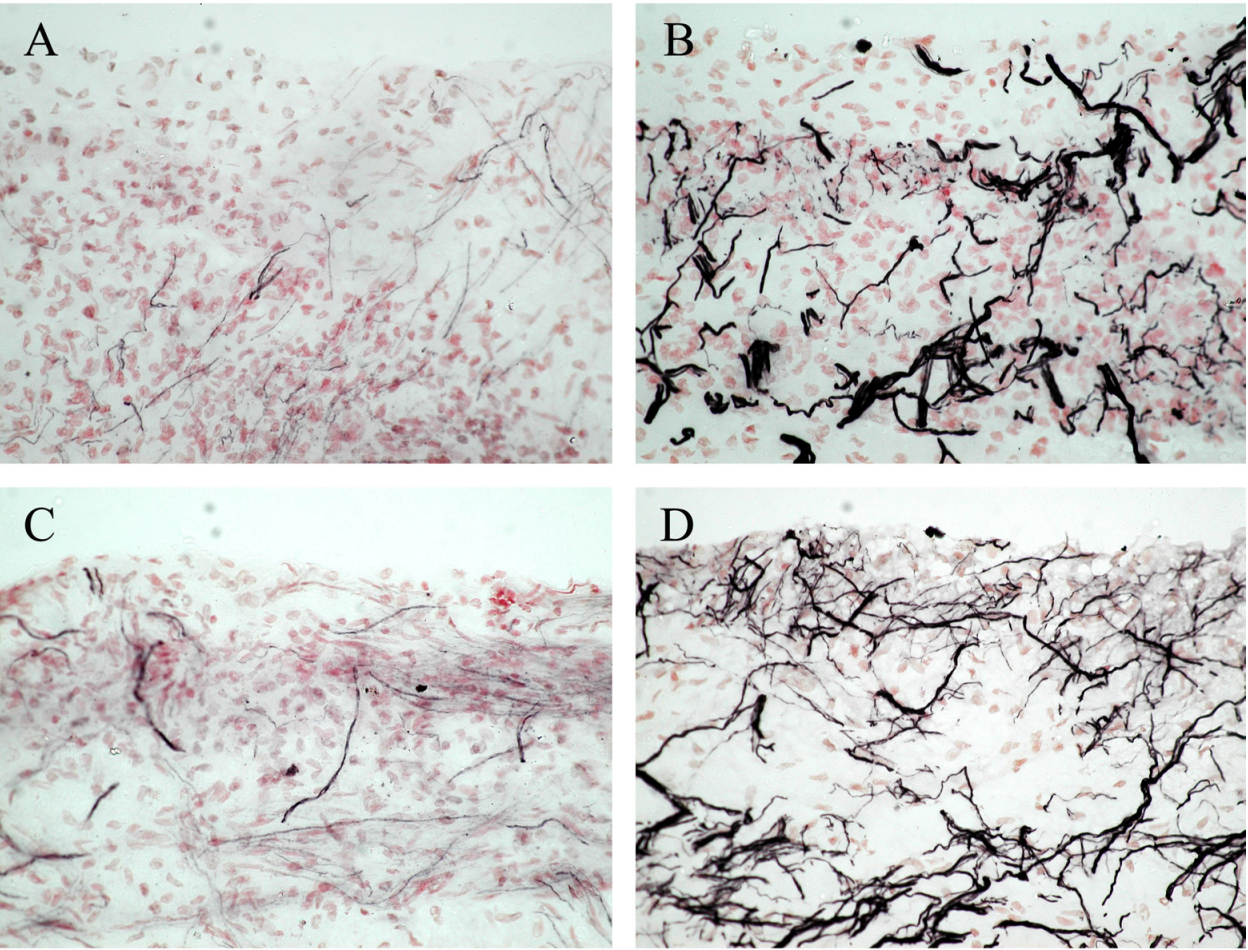
Immunoreactive nerve fibres in non-painful (left column) and painful (right column) human tooth pulp sections, in pulp from painful teeth within the subontoblastic plexus region 'boxed' in Figure 1. Staining with antibodies to Na_v_1.8 (A and C) and neurofilament (B and D). Arrows indicate Na_v_1.8 immunoreactive nerve fibres. Magnification × 40

Figure 1 shows Neurofilament staining of the pulpal horn of a molar tooth highlighting the relationship of the subodontoblastic plexus (boxed area) to the deeper relatively poorer innervated coronal pulp at low magnification. Immunostaining demonstrated the presence of large numbers of nerve fibres within human tooth pulp that were immunoreactive for neurofilament (Fig. 2A and B). A subset of nerve fibres was also immunostained with the Na_v_1.8 antibody (Fig. 2C and D) in both non-painful and painful pulp groups. Specificity of the Na_v_1.8 antibody was demonstrated by methods described for this antibody by us in human DRG [9], including omission of primary antibody and pre-incubation with peptide.

**Figure 3:**
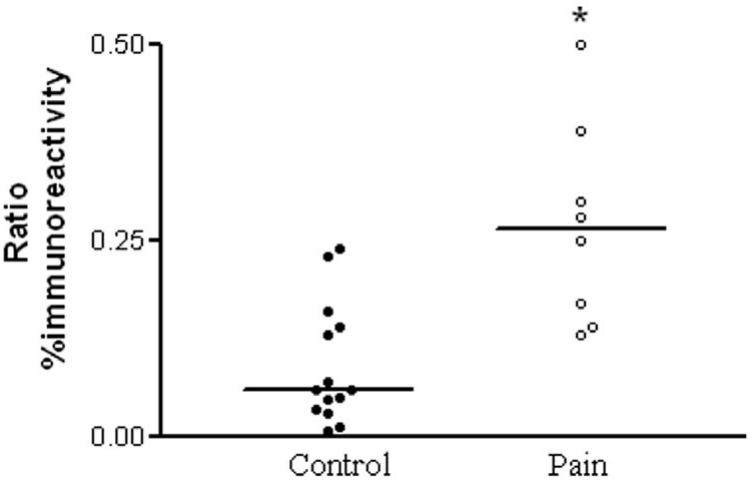
Scattergram of Na_v_1.8 to Neurofilament ratio of the % area in control and painful tooth pulp. The median value is indicated * P < 0.005.

By image analysis, neurofilament fibre median % area (range) in non-painful tooth pulp was 13.94 (3.05–22.05), and in painful tooth pulp was 18.21 (9.11–27.88); there was trend for an increase, but this was not statistically significant. There was a significant change of the corresponding Na_v_1.8 % area in non-painful 0.68 (0.13–2.90) compared to painful tooth pulp 5.855 (1.3–7.52), *P *< 0.005. There were also significantly more fibres immunostaining for Na_v_1.8 in relationship to neurofilament positive fibres in the painful pulp, compared with those without pain (Fig 3). The median Na_v_1.8 to Neurofilament % area ratios were for non-painful 0.059 (0.006–0.24), and for painful 0.265 (0.13–0.5), *P *< 0.005. A degree of inflammation was seen in all painful pulp samples.

## Discussion

Neurofilament positive fibres have previously been reported in human dental pulp, including fine unmyelinated fibres in the sub odontoblastic layer [11,12]. However, thus far there have been few investigations of the expression of ion channels in the human dental pulp. We have demonstrated for the first time that numerous Na_v_1.8-immunoreactive nerve fibres are present in human dental pulp in the subodontoblastic layer, and the Na_v_1.8-immunoreactive fibres were increased in the presence of caries-induced painful pulpitis. While there was a trend for increased neurofilament fibres in painful dental pulp, this was not statistically significant. An increase of nerve fibres within inflamed dental pulp, possibly due to nerve sprouting, has been previously reported in the rat dental pulp after dentine injury [13], and other studies report an increased neuropeptide expression and sprouting in the human infected dental pulp [14,15]. In contrast to our findings for Na_v_1.8 in this study, our previous study of TRPV1 and P2X3 receptors showed no significant change in painful dental human dental pulp [16].

The presence of Na_v_1.8-immunoreactive neurons identified in the subodontoblastic layer implies that these receptors may be involved in signal transduction at the pulp-dentine junction. It is known that sensory neurones and odontoblasts exist in close proximity, but no synaptic or electrical connections have been identified [2,12] postulated that the odontoblast-neuron connection may be neurochemical. Infection or injury to the pulpal tissues may result in inflammation, resulting in increased expression of substance P, CGRP and collateral nerve sprouting, which are regulated by nerve growth factor (NGF), which also regulates Na_v_1.8 expression by sensory neurons [7]; NGF is itself increased in inflamed pulpal tissues [17]. The sampling of healthy non-painful pulp from partially erupted third molars, though developmentally mature, may not be representative of fully erupted molar pulps. Fibre numbers and receptor expression may change after eruption of the tooth [2]; it remains unknown whether Na_v_1.8-immunoreactivity varies with eruption or maturity of teeth.

Although our cross reactivity and pre-absorption studies showed specificity of the antibodies used, the data should be interpreted with caution. Immunostaining of nerves appears to be axoplasmic in this study, and not at the nodes of Ranvier. Recently [18] Henry and colleagues have reported Na_v_1.8-immunoreactivity associated with the nodes of Ranvier in the radicular human tooth pulp. The difference in localisation between the studies could reflect characteristics of the antibodies and immunostaining methods, and further studies, including different techniques and functional assays, are necessary. The relative expression of Na_v_1.8-immunoreactive fibres to neurofilament positive fibres is low – this may also be due to the affinity of the antibody used in this study, or reflect the expression of this ion channel in a small sub-set of nerve fibres. The specific type of fibre expressing Na_v_1.8, the distribution of Na_v_1.8 throughout the human dental pulp, and the longitudinal changes in the Na_v_1.8-immunoreactivity caused by pulpal inflammation, all require further study.

## Conclusion

In conclusion, nerve fibres in dental pulp from patients with dental pain showed significantly more Na_v_1.8-fibres as a proportion of neurofilament positive fibres. As Na_v_1.8 has been implicated in neuropathic pain, its expression by nerve fibres within human tooth pulp may contribute to the pathophysiology of dental pain. Further studies of the time-course of the disease, and severity of pain and/or inflammation, are necessary to elucidate the role and regulation of Na_v_1.8 ion channels in the pathophysiology of trigeminal pain. Na_v_1.8 represents a target for novel analgesic agents.

## Competing interests

The author(s) declare that they have no competing interests.

## Authors' contributions

TR performed all the surgical procedures, extracted the tooth pulp and helped write the paper. YY participated in the immunohistochemistry, analysis of data and drafted the manuscript. CP, ST and CB were responsible for the design and production of the Na_V_1.8 antibodies used, help with interpretation of the data, and writing the manuscript. CB participated in the conception of the study, development of antibodies, and interpreting the data. PA conceived the study and participated in its design and coordination, interpretation and completion of the manuscript. All authors read and approved the manuscript.

## Pre-publication history

The pre-publication history for this paper can be accessed here:


